# Use of ultrasonography in confirming the malposition of the i-gel in pediatric patients: a prospective observational study

**DOI:** 10.55730/1300-0144.5401

**Published:** 2022-03-19

**Authors:** Faruk ÇİÇEKCİ, Mehmet Selçuk ULUER, Mehmet SARGIN, Emine ASLANLAR, İnci KARA, Muslu Kazım KÖREZ

**Affiliations:** 1 Department of Anesthesiology and Reanimation, Faculty of Medicine, Selçuk University, Konya, Turkey; 2 Department of Biostatistics, Faculty of Medicine, Selçuk University, Konya, Turkey

**Keywords:** Airway management, fiberoptic examination, leakage test, ultrasonography

## Abstract

**Background/aim:**

This study was designed to observe and compare the performance of fiberoptic laryngeal (FOL) view, leakage test (LT) and ultrasonography (USG) usage in detecting i-gel position in pediatric patients.

**Materials and methods:**

One hundred ten consecutive children were included in this single-armed prospective observational study. After anesthetic induction, i-gel placement confirmed using FOL and LT was evaluated using USG in three planes. According to our scoring system, acceptable and unacceptable grades (FOL, LT and USG) were determined to describe placement. Sensitivity and specificity were determined by comparing USG performance with the other two tests.

**Results:**

Ultrasonography was found a sensitivity of 20% positive predictive value (PPV) for unacceptable i-gel placement according to FOL grade and a sensitivity of 37.04% with a 100% PPV according to LT grade. USG was found a specificity of 91.84% negative predictive value (NPV) of 91.84% for acceptable i-gel placement according to FOL grade and the NPV specificity of 100% with a 82.65% NPV according to LT grade.

**Conclusion:**

Ultrasonography demonstrated a very good diagnostic performance in the detection of optimal i-gel placement according to both FOL and LT. However, both FOL and LT showed poor diagnostic performance compared to USG in demonstrating i-gel malposition.

## 1. Introduction

The use of i-gel, as a relatively new supraglottic airway device (SAD) released in 2007, has become popular for children undergoing surgery with general anesthesia because it does not require muscle relaxation. It is important to place an i-gel in the optimal position to ensure adequate ventilation and avoid complications such as mucosal injury, glottic ptosis, and possible aspiration and gastric insufflation [1,2]. Successful insertion is usually assessed using a capnogram with an end-tidal carbon dioxide (EtCO_2_) value, appropriate chest elevation, no more than 20 cmH_2_O peak inspiratory pressure and the absence of audible oropharyngeal leakage [2]. Children have broad tongues and a drooping epiglottis. Also, their larynx is higher and more anterior than in adults. This difference in airway anatomy may affect the correct placement of SADs [3]. Although fiberoptic laryngeal (FOL) view is considered the preferred verification tool for direct visualization of the glottis, it has also been reported to be a superior technique for detecting the malposition of SADs [4–6]. Some studies of SAD position using FOB have reported that a proportion of children need repositioning of smaller SADs (12.8%–49%) despite adequate ventilation [2,7,8]. Ultrasonography (USG), which is a noninvasive, simple and portable technology, has recently come into use in evaluating airway management even in upper airway anatomy impaired due to pathology or trauma [9,10]. However, although several publications on ultrasonography confirming the optimal location of various SADs have been reported recently, USG publications examining its malposition have not been reported [11,12].

The aim of this study was to compare the FOL image and leakage test (LT) values used in the placement of i-gels with USG findings in pediatric patients and to demonstrate the performance of USG in confirming i-gel malposition.

## 2. Methods

This single-arm prospective observational study was reviewed and approved by the Selçuk University Faculty of Medicine the Local Ethics Committee (14.10.2020.2020/19) and recorded in the Clinical Trials Registry (ClinicalTrials.gov Identifier: NCT04652466). After obtaining written informed consent from the parents of the participants, 110 patients with American Society of Anesthesiologists (ASA) status I–II undergoing elective pediatric surgery and had I-gels placed were enrolled in the study. Patients with head and neck anatomic malformations and airway congenital defects were excluded from the study.

Midazolam 0.5 mg kg^−1^ was administered orally 20 min before surgery for routine premedication to all patients. The patients were placed on the operating table in the supine position. All patients were monitored with standard monitoring (electrocardiogram, noninvasive blood pressure, oxygen saturation, EtCO_2_, peak airway pressure), after a minimum of 2 min of preoxygenation, inhalational induction was performed with 6%–7% sevoflurane at the beginning of general anesthesia induction. IV access was provided, and according to standard procedures, propofol 3.5 mg/kg, fentanyl 1 mcg/kg iv and remifentanil 0.1 mcg/kg/min infusion was administered. The i-gel size was selected according to the manufacturer’s recommendations. Adequate anesthesia depth was confirmed by loss of eyelash reflex, symmetrical small pupils, and absence of swallowing, and i-gel was gently guided along the hard palate by the first anesthesiologist until resistance was felt, with the i-gel (Intersurgical Ltd., Wokingham, Berkshire, UK) opening facing the applicator. Patients were mechanically ventilated with an EtCO_2_ value between 35 and 45 mmHg with volume-controlled ventilation, at 3 L/min of fresh gas flow, 6–8 ml/L tidal volume, and 18 to 24 breaths/min frequency. After 5 ventilator cycles, Ppeak was recorded. If the patient did not tolerate the insertion of the i-gel, an extra dose of fentanyl up to a maximum of 0.5 mcg/kg was administered. However, if the insertion attempt was still not tolerated, or if the capnography curve was not obtained and there was an audible leakage, the technique was considered to be unsuccessful, and another supraglottic SAD was then placed in the airway. If this change was not tolerated, endotracheal intubation was initiated by administering a neuromuscular blocker and these were excluded. All patients were evaluated by a total of four experienced independent anesthetists who performed intubation, FOL, LT, and USG examinations.

### 2.1. Fiberoptic laryngoscopy examination

**Figure 1:**
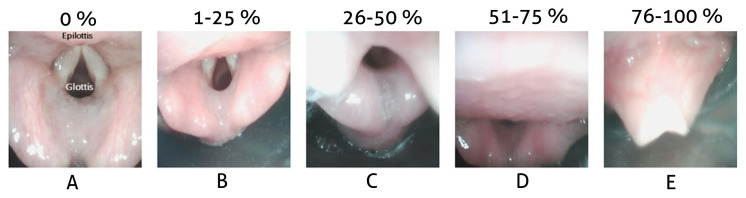
Fiberoptic appearance of the glottis: Percentage and categories of glottic opening covered by the epiglottis. A–C indicates that the epiglottis visibility is lesser than 50% and the epiglottis is positioned correctly. D and E indicates that the epiglottis visibility is more than 50% and the epiglottis is misplaced.

A fiberoptic laryngoscope was passed between the Y-connector and the i-gel to obtain the FOL by the second anesthesiologist. The image acquired by the FOL advanced from i-gel was categorized as described by Campbell et al. [13]. The optimal placement was defined as epiglottis visibility of less than 50% (Figures 1A–1C) where the epiglottis did not enter the airway through the i-gel; acceptable placement was recorded as FOL-A (fiberoptic laryngoscopy acceptable). Categories E and F were defined as more than 50% epiglottis visibility where the epiglottis entered the airway; unacceptable insertion was recorded as FOL-U (fiberoptic laryngoscopy unacceptable) (Figures 1D and 1E) (Table 1).

### 2.2. Leakage test (LT) examination

LT was determined by temporarily ceasing ventilation and closing the adjustable pressure-limiting valve with a fresh gas flow of 3 L min^−1^ until the airway pressure reached a steady state and a leakage sound was heard by the third anesthesiologist. The leakage pressure was not allowed to exceed 40 cm H_2_O. The LT grading system was based on the study of Theiler et al. [14]. Acceptable LT (LT-A) was recorded as peak inspiratory pressure (PIP) ≤ 26 cm H2O, bilateral chest lift and square wave capnogram formation, no square wave capnogram, an audible leakage. PIP ≥ 27 cm H_2_O were recorded as unacceptable LT (LT-U) (Table 1).

### 2.3. Evaluation of i-gel placement using USG

**Figure 2:**
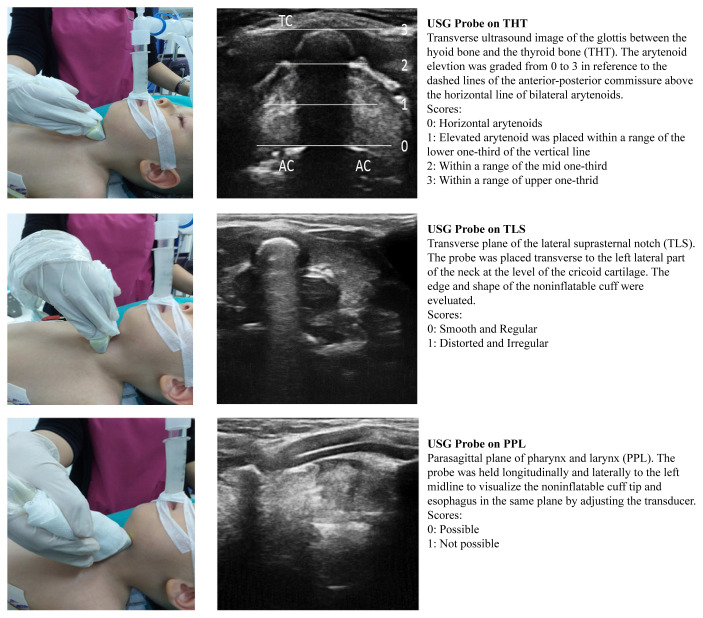
Figure showing the ultrasound airway examination and ultrasound examination scoring system in three different planes. AC = arytenoid cartilages, TC= thyroid cartilage.

USG examination of the neck was performed by the fourth anesthesiologist using an Esaote 8–18–MHz linear probe (Mylab 30 gold, Esaote, Toscana, Italy). Imaging was performed in three planes, and the USG score was assigned to the deployment view using a modification of the criteria proposed by Song et al. [15] (Figure 2). The viewing planes and scoring system used were as follows:

1. Transverse plane between the hyoid bone and thyroid bone (THT). The probe was placed transversely between the hyoid bone and thyroid cartilage, and the noninflatable cuff shadow was seen in this view.

Score – Symmetrical: 0; Asymmetrical: 1 (1, elevated arytenoid was placed within a range of lower one-third of vertical line; 2, within a range of mid one third; 3, within a range of the upper one-third) (Figure 2).

2. Transverse plane of the lateral suprasternal notch (TLS). The probe was placed transverse to the left lateral part of the neck at the level of the cricoid cartilage. The edge and shape of the noninflatable cuff were evaluated.

Score - Smooth and Regular: 0; Distorted and Irregular: 1 (Figure 2).

3. Parasagittal plane of pharynx and larynx (PPL). The probe was held longitudinally and laterally to the left midline to visualize the noninflatable cuff tip and esophagus in the same plane by adjusting the transducer. Score – Possible: 0; Not possible: 1 (Figure 2).

The USG glottic images obtained were recorded for later evaluation. Grading was made according to the score points obtained. If the total score obtained was 0 or 1, the image was defined as grade I and was considered to show an acceptable location (USG-A). If the total score was between 2 and 5, the image was defined as grade II and considered to indicate an unacceptable location (USG-U) (Table 1).

If adequate ventilation was not provided, correction of i-gel was attempted or another method (i-gel size changed, SAD insertion or ETI applied). The i-gel was removed when patients regained consciousness at the end of the method.

### 2.4. Statistical analysis

The study by Kim et al. [11] on the difference between USG and FOL in the incidence of i-gel malposition was accepted as a reference. The incidence of i-gel malposition with FOL was 50% and 43% on USG. Assuming a 5% significance level (α = 0.05) and power of 80% (= 0.20) to detect a 7% absolute difference in the incidence of i-gel malposition with USG and FOL, a sample size of 102 patients was required. The sample size allowing for possible data loss was allocated as 110 patients.

All statistical analyses were performed using the online *R* 3.6.0 program (https://www.r-project.org). Data are presented as mean ± standard deviation (range: minimum–maximum) or numbers (*n*) and percentages (%). Kappa (*κ*) coefficient analysis was used to test the agreement between FOL, USG, and LT grades. The diagnostic performance was tested with sensitivity, specificity, positive and negative predictive value (PPV and NPV, respectively), and accuracy. The sensitivity was the percentage of USG grades 2 to 5 and LT grades higher than ≥27 that were correctly identified as i-gel malposition grades 4 to 5 using FOL. The specificity was the percentage of USG grades 0 to 1, and LT grades lower than ≤26 that were correctly identified as i-gel malposition grades 1 to 3. The PPV was the percentage of i-gel placement grades 4 to 5 when USG arytenoid image grades were 3 to 5, and LT grades were higher than ≥ 27. The NPV was the percentage of i-gel placements 1 to 3 when the USG arytenoid image was 0 to 1 and LT grades were lower than ≤26. Accuracy was the percentage of concordance between 2 devices in terms of grades. The confidence interval of the abovementioned parameters was calculated using the Clopper–Pearson method. Chi-square and *Z*-tests were used to compare the rate ratios of malposition between FOL, USG, and LT methods. Moreover, the McNemar test was used to compare the proportions of the methods. A *p-*value of less than 0.05 was considered statistically significant.

## 3. Results

**Figure 3:**
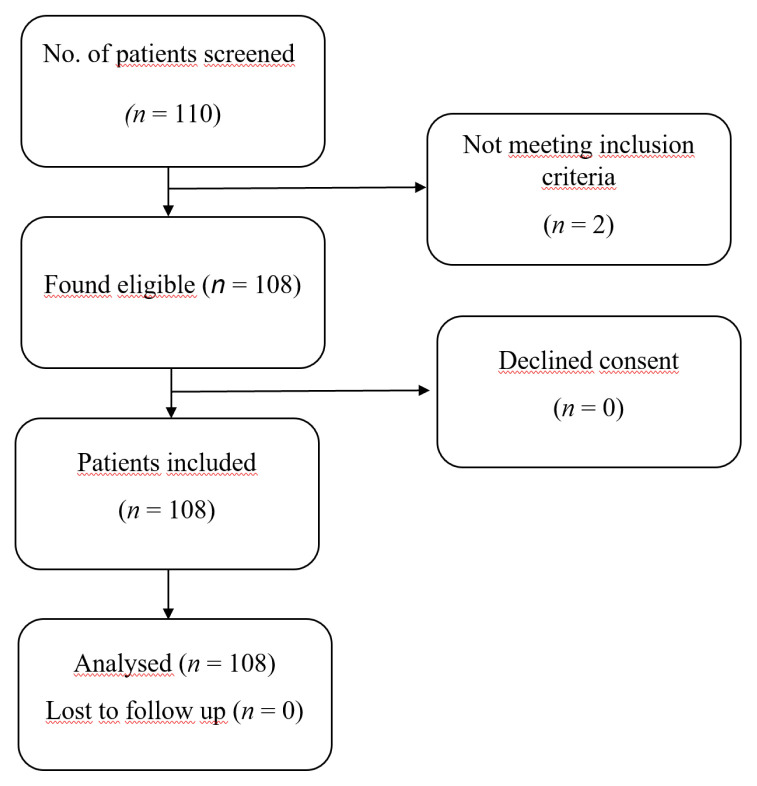
CONSORT flowchart.

A total of 110 patients underwent elective pediatric surgery between February 2021 and December 2020. A total of 2 patients were excluded for not meeting inclusion criteria. Thus, 108 patients were enrolled. Detailed information on enrollment of patients into the study is depicted in the CONSORT flow diagram in Figure 3. The demographic and clinical characteristics of the children are given in Table 2. A total of 108 children who had a mean age of 3.64 ± 2.29 (range, 1–10) years, mean BMI of 15.23 ± 3.05 (range, 3.79–25.14), were included in the study. The i-gel was successfully inserted at the first attempt in 98 (90.7%), at the second attempt in nine (8.3%), and at the third attempt in one (0.9%) child. The mean EtCO_2_ was 39.82 ± 4.43 (range, 30–45), the mean SpO2 was 98.77 ± 1.09 (range, 96–100), and the mean Ppeak was 12.03 ± 3.19 (range, 6–23). There was no significant difference in Ppeak between the FOL grades (*p* = 0.144), but the mean Ppeak was higher in unacceptably positioned i-gels compared with acceptably positioned i-gels in both USG (16.20 ± 4.87 vs. 11.60 ± 2.66, *p* < 0.001) and LT (14.52 ± 4.11 vs. 11.19 ± 2.32, *p* < 0.001) grades (Table 2).

In the FOL grade, 63 children (58.3%) were found to be grade 1, 28 (25.9%) were grade 2, 7 (6.5%) were grade 3, 7 (6.5%) were grade 4, and 3 (2.8%) were grade 5. FOL grade >3 was seen in 10 out of 108 (9.26%) (range, 4.53%–16.37%) patients where i-gel was defined as unacceptable, and FOL grade ≤ 3 was seen 98 (90.74%) (range, 83.63%–95.74%) patients where the i-gel was defined as acceptable (Table 3).

In the LT, 81 out of 108 (75%, 95% CI: 65.75%–82.83%) children whose LT scores were lower than 28 were found to be acceptable, and 27 (25%, 95% CI: 17.17%–34.25%) children whose LT score was ≥ 27 were found to be unacceptable. The mean LT score of the children was 24.51 ± 6.40 (range, 13–39) (Table 3).

In the USG examination grade, 86 children (79.6%) were found to be grade 0, 12 (11.1%) grade 1, 7 (6.5%) grade 2, and 3 (2.8%) grade 3. USG grade > 1 was seen in 10 out of 108 children (9.26%, 95% CI: 4.53%–16.37%) in whom i-gel placement was defined as unacceptable, and USG grade ≤ 1 was seen 98 (90.74%, 95% CI: 83.63%–95.74%) children, where i-gel was defined as acceptable (Table 3).

The rate of i-gel displacement was similar between FOL grades and USG grades (*p* > 0.999), but this incidence was lower in USG grade compared with LT grade (*p* = 0.022) (Table 3).

Ninety out of 108 children had acceptable USG and FOL grades in i-gel placement. Two out of 108 children had acceptable USG and FOL grades in i-gel placement. Eighty-one out of 108 children had acceptable USG and LT grades in i-gel placement. Ten out of 108 children had acceptable USG and FOL grades in i-gel placement. Of the 98 i-gel placements that were positioned acceptably according to the FOL grade, 90 were successfully positioned using USG. In addition, of the 10 unacceptable i-gel placements that were positioned according to FOL grade, two were successfully positioned using USG. Of the 81 acceptable i-gel placements that were positioned according to FOL grade, 81 were successfully positioned using USG. Of the 27 unacceptable i-gel placements that were positioned according to the FOL grade, 10 were successfully positioned using USG (Table 4).

Regarding the diagnostic performance of USG in terms of FOL and LT grade, USG was found to have a sensitivity of 20% (2.52%–55.61%), with a PPV of 20% (5.77%–50.50%) to detect an unacceptable i-gel placement according to FOL grade. The specificity was 91.84% (84.5%–96.41%), with an NPV of 91.84% (89.14%–93.91%) to detect an acceptable i-gel placement according to FOL grade. The accuracy was 85.19% (77.06%–91.29%). USG was found to have a sensitivity of 37.04% (19.40%–57.63%), with a PPV of 100% (69.2%–100%) to detect an unacceptable i-gel placement according to LT grade. The specificity was 100% (95.55%–100%), with an NPV of 82.65% (78.11%–86.42%) to detect an acceptable i-gel placement according to LT grade. The accuracy was 84.26% (76%–90.55%). The kappa value was 0.118 ± 0.096 between USG and FOL grade, and there was no statistically significant agreement between USG and FOL grade (*Z* = 1.23, *p* = 0.218). However, the kappa value was 0.468 ± 0.081, which suggests a moderate agreement between USG and LT grade, and this kappa value is significantly different from zero (*Z* = 5.75, *p* < 0.001) (Table 4).

## 4. Discussion

Fiberoptic laryngoscope view and LT grades were compared with USG grades in i-gel positioning. It was shown that USG was compatible with FOL and LT in showing the optimal i-gel placement, but its diagnostic performance was poor for malpositioned i-gels.

Although the most commonly used test to evaluate the position of SADs is the LT, its use in cases of laryngospasm or bronchospasm may be restricted, regardless of the position of the SADs in the oropharynx [14]. Therefore, imaging the airway with fiberoptic is the gold standard in confirming the location of SADs and avoiding airway-related adverse events [16].

However, although FOL assessment is an important assessment tool, it may not be used routinely because it is technically demanding and an invasive procedure. Recently, there has been a great interest in the use of USG in airway management, which allows uninterrupted airway management as well as being safe, fast, portable, and repeatable. Previously limited to airway evaluation, it now finds use in real-time dynamic airway management [9,10].

The first report on USG evaluation for proper placement of SADs was published by Kim et al. In their study [11], they compared USG images of positional changes in arytenoids before and after insertion of classical LMA in 100 pediatric patients. USG was found to have a sensitivity of 93% and specificity of 82% (accuracy was 87% to detect a rotated LMA) [16.] In the LMA study of Zhou et al. clinical findings, FOL and USG were compared, and it was suggested that USG evaluation was superior to other techniques to confirm proper LMA placement [17]. In the last few years, several studies have been conducted in which USG verification of the position of SADs was evaluated and compared with other methods, and USG was found as an appropriate tool for confirming SAD placement [15,18].

Although both USG and fiberoptic evaluation were based on the evaluation of the anatomic relationship between the i-gel noninflating cuff and laryngeal entrance, there were some inequalities in our results. In our study, the specificity was 91.84% with an NPV of 91.84% to detect an acceptable i-gel placement according to the FOL grade of USG, regarding the diagnostic performance of USG in terms of FOL and LT. The specificity was 100% with an NPV of 82.65% to detect an acceptable i-gel placement based on the LT grade of USG. In other words, USG has high concordance with FOL and LT when the i-gel position is optimal. However, USG was found to have a sensitivity of 20%, with a positive predictive value (PPV) of 20% to detect unacceptable i-gel placement according to FOL grade. This result, on the other hand, shows that USG has a poor concordance in detecting i-gel malposition when compared to FOL and LT. The reason for this may be that, especially in young children, even if ventilation is sufficient, it may give an unacceptable FOL appearance, although it gives an acceptable appearance on USG due to its more anterior and highly located larynx, floppy epiglottis, larger tongues, small-sized laryngeal, and oropharyngeal anatomy [19].

Children are known to be more susceptible to complications than adults from the use of SADs [20]. The most common complications are minor and transient and include sore throat, difficulty swallowing, and hoarseness. Serious complications are very scarce and include oropharyngeal trauma, nerve damage or stomach contents aspiration [21]. In our study, broncho/laryngospasm and bradycardia were observed in only one patient (0.9%). The results were consistent with the literature [21,22].

The strength of the study was the employment of separate anesthesiologists for anesthesia induction, FOL, USG imaging, and LT to overcome observer bias. In order to avoid observer bias in the USG imaging, the anesthesiologist was trained in airway imaging of SADs for about 1 week by the radiology department. Another is that we used Song et al.’s 15 system to rule out observer bias and obtain a standard view in a performance-dependent and subjective assessment [23] such as in a USG examination.

Nevertheless, there were some limitations in our study. First, we used i-gel, a second-generation SGA with a silicone body and noninflatable cuffs, but we cannot generalize our results to other structurally different second-generation SGAs as the degrees of intraoral sealing may be affected.

## 5. Conclusion

Ultrasound examination of the airway is strongly correlated with FOL and LT grade when determining the position of an i-gel and is highly compatible with acceptable positioning. USG has demonstrated very good diagnostic performance in the acceptable placement of i-gels according to both FOL and LT. However, USG showed poor diagnostic performance in confirming malpositioned i-gels.

## Figures and Tables

**Table 1:** Grading according to FOL, USG and LT.

FOL grade	
FOL-A	The categories A-B-C
FOL-U	The categories D-E

USG grade	
USG-A	The scores 0–1
USG-U	The scores 2–5

Leakage test grade	
LT-A	Leakage test ≤ 26 cmH_2_O
LT-U	Leakage test ≥ 27 cmH_2_O

FOL: fiberoptic laryngoscopy, USG: ultrasonography, LT: leakage test, A: acceptable, U: unacceptable.

**Table 2:** Demographic and clinical characteristics of the patients.

Characteristics	*n* = 108
Age (years)	3.64 ± 2.29 (1–10)
BMI	15.23 ± 3.05 (3.79–25.14)
ASA (1/2)	100 (92.5) / 8 (7.4)
I-Gel no (1.0/1.5/2.0/2.5/3.0)	1 (0.9) / 30 (27.8) / 58 (53.7) / 17 (15.7) / 2 (1.9)
I-Gel insertion attempt (1st/2nd/3th)	98 (90.7) / 9 (8.3) / 1 (0.9)
LMA insertion	0 (0)
ETI insertion	0 (0)
Peak airway pressure	12.03 ± 3.19 (6–23)
EtCO_2_	39.82 ± 4.43 (30–45)
SpO_2_	98.77 ± 1.09 (96–100)

Data are given as mean ± SD (min – max) or number (%).SD: standard deviation, ASA: the American Society of Anesthesiologists, ETI: endotracheal intubation.

**Table 3:** Distribution of i-gel position according to USG, LT, and FOL test.

Test results	*n* = 108

FOL image (1/2/3/4/5), *n* (%)	63 (58.3) / 28 (25.9) / 7 (6.5) / 7 (6.5) / 3 (2.8)

FOL grade, *n* (%)	
FOL-A	98 (90.7)
FOL-U	10 (9.3)

THT (0/1/2), *n* (%)	86 (79.6) / 17 (15.7) / 5 (4.6)

TLS (0/1), *n* (%)	102 (94.4) / 6 (5.6)

PPL (0/1), *n* (%)	106 (98.1) / 2 (1.9)

USG score (0/1/2/3), *n* (%)	86 (79.6) / 12 (11.1) / 7 (6.5) / 3 (2.8)

USG grade, *n* (%)	
USG-A	98 (90.7)
USG-U	10 (9.3)

Leakage test score, mean ± SD (min – max)	24.51 ± 6.40 (13–39)

Leakage test grade, *n* (%)	
LT-A	81 (75)
LT-U	27 (25)

Data are given as mean ± SD (min – max) or number (%).FOL: fiberoptic laryngoscopy, THT: transverse plane between hyoid bone and thyroid bone, TLS: transverse plane of lateral suprasternal notch, PPL: parasagittal plane of pharynx and larynx, USG: ultrasonography.

**Table 4:** Frequency table and agreement statistics of LT grade, USG, and FOL grade.

	FOL grade		Leakage test grade		

	FOL-A	FOL-U	LT-A	LT-U	Total (%)

USG grade, *n* (%)					
USG-A	90 (91.8)	8 (80)	81 (100)	17 (63)	98 (90.7)
USG-U	8 (8.2)	2 (20)	0 (0)	10 (37)	10 (9.3)
Total (%)	98 (90.7)	10 (9.3)	81 (75)	27 (25)	108

McNemar test, *p*-value	*χ*^2^ = 0.063, *p* = 0.803		*χ*^2^=15.058, *p* < 0.001		

Agreement statistics					
*κ* ± SE	0.118 ± 0.096		0.468 ± 0.081		
*Z*(*κ*), *p*-value	*Z* = 1.23, *p* = 0.218		*Z* = 5.75, *p*<0.001		
Proportion A agreement	91.84%		90.50%		
Proportion U agreement	20%		54.05%		

Diagnostic measures (%, 95% CI) for unacceptable					
Sensitivity	20 (2.52–55.61)		37.04 (19.40–57.63)		
Specificity	91.84 (84.55–96.41)		100 (95.55–100)		
PPV	20 (5.77–50.50)		100 (69.2–100)		
NPV	91.84 (89.14–93.91)		82.65 (78.11–86.42)		
Accuracy	85.19 (77.06–91.29)		84.26 (76–90.55)		

Incidence rate for U in USG (%, 95% CI) (9.26 % (17.17–34.25)					
Incidence rate for A	83.33% (74.94–89.81)		75% (65.75–82.83)		
Incidence rate for U	9.26 % (17.17–34.25)		25 % (17.17–34.25)		

Comparison of incidence rate for U					
with *Z*-test	*p* > 0.999		*Z* = 10.1, *p* < 0.001		
with *χ*^2^ test	*p* > 0.999		*χ*^2^ = 9.381, *p* = 0.022		

Relationship between methods (for numerical data)					
Spearman’s *rho*	*r_s_* = 0.203, *p* = 0.035		*r_s_* = 0.345, *p* < 0.001		
Kendall’s *τ* B	*τ* = 0.191, *p* = 0.032		*r_s_* = 0.345, *p* < 0.001		

FOL: fiberoptic laryngoscopy, USG: ultrasonography, A: acceptable, U: unacceptable, *κ*: kappa statistics, SE: standard error of *κ*, *Z*(*κ*): *Z* test for *κ*, PPV: positive predictive value, NPV: negative predictive value.

## References

[1] Levitan RM Kinkle WC Initial anatomic investigations of the I-gel airway: a novel supraglottic airway without inflatable cuff Anaesthesia 2005 60 10 1022 6 10.1111/j.1365-2044.2005.04258.x 16179048

[2] Von Ungern-Sternberg BS Wallace CJ Sticks S Erb TO Chambers NA Fibreoptic assessment of paediatric sized laryngeal mask airways Anaesthesia and Intensive Care 2010 38 1 50 54 10.1177/0310057X1003800110 20191777

[3] Shimbori H Ono K Miwa T Morimura N Noguchi M Hiroki K Comparison of the LMA-ProSeal and LMA-Classic in children British Journal of Anaesthesia 2004 93 4 528 531 10.1093/bja/aeh238 15298876

[4] Brimacombe J Keller C Fullekrug B Rosenblatt W Dierdorfet SF A Multicentre study comparing the ProSeal with the Classic laryngeal mask airway in anesthetized, nonparalyzed patients Anesthesiology 2002 96 2 289 295 10.1097/00000542-200202000-00011 11818758

[5] Keller C Brimacombe J Mucosal pressure and oropharyngeal leak pressure with the ProSeal versus laryngeal mask air way in anaesthetized paralysed patients British Journal of Anaesthesia 2000 85 2 262 266 10.1093/bja/85.2.262 10992836

[6] Cook TM Nolan JP Verghese C Lees M Millar JM Baskett PJF Randomized crossover comparison of the ProSeal with the classic laryngeal mask air way in unparalysed anaesthetized patients British Journal of Anaesthesia 2002 88 4 527 533 10.1093/bja/88.4.527 12066729

[7] Ghai B Ram J Makkar JK Wig J Fiber-optic assessment of LMA position in children: a randomized crossover comparison of twotechniques Pediatric Anaesthesia 2011 21 11 1142 1147 10.1111/j.1460-9592.2011.03632.x 21689206

[8] Soh CR Ng AS Laryngeal mask airway insertion in paediatric anaesthesia: comparison between the reverse and standard techniques Anaesthesia and Intensive Care 2001 29 5 515 519 10.1177/0310057X0102900512 11669434

[9] Kundra P Mishra SK Ramesh A Ultrasound of the airway Indian Journal of Anaesthesia 2011 55 5 456 462 10.4103/0019-5049.89868 22174461 PMC3237144

[10] Kristensen MS Ultrasonography in the management of the airway Acta Anaesthesiologica Scandinavica 2011 55 10 1155 1173 10.1111/j.1399-6576.2011.02518.x 22092121

[11] Kim J Kim JY Kim WO Kil HK An ultrasound evaluation of laryn-geal mask airway position in pediatric patients: an observational study Anesthesia and Analgesia 2015 120 2 427 432 10.1213/ANE.0000000000000551 25545750

[12] Arican S Pekcan S Hacibeyoglu G Yusifov M Yuce S Uzun ST The place of ultrasonography in confirming the position of the laryngeal mask airway in pediatric patients: an observational study Revista Brasileira de Anestesiologia 2021 71 5 523 529 10.1016/j.bjane.2020.12.014 PMC9373655 34537123

[13] Campbell RL Biddle C Assudmi N Campbell JR Hotchkiss M Fiberoptic assess-ment of laryngeal mask airway placement: blind insertionversus direct visual epiglottoscopy Journal of Oral and Maxillofacial Surgery 2004 62 9 1108 1116 10.1016/j.joms.2003.10.014 15346362

[14] Theiler L Kleine-Brueggeney M Urwyler N Graf T Luyet C Greif R Randomized clinical trial of the i-gel and Magill tracheal tube or single-use ILMA and ILMA tracheal tube for blind intubation in anaesthetized patients with a predicted difficult airway British Journal of Anaesthesia 2011 107 2 243 250 10.1093/bja/aer102 21652615

[15] Song K Yi J Liu W Huang S Huang Y Confirmation of laryngeal mask airway placement by ultrasound examination: A pilot study Journal of Clinical Anaesthesia 2016 34 638 466 10.1016/j.jclinane.2016.06.019 27687463

[16] Abraham A Gold standards and anaesthesia Indian Journal of Anaesthesia 2013 57 2 207 209 10.4103/0019-5049.111876 23825831 PMC3696279

[17] Zhou Z Xia C Wu M Yu L Yan GZ Comparison of three methods for the confirmation of laryngeal mask airway placement in female patients undergoing gynecologic surgery Ultrasound in Medicine and Biology 2015 41 5 1212 1220 10.1016/j.ultrasmedbio.2014.12.002 25748523

[18] Wojtczak JA Cattano D Laryngo-tracheal ultrasonography to confirm correct endotracheal tube and laryngeal mask airway placement Journal of Ultrasonography 2014 14 59 362 366 10.15557/JoU.2014.0037 26672974 PMC4579715

[19] Okuda K Inagawa G Miwa T Hiroki K Influence of head and neck position on cuff position and oropharyngeal sealing pressure with the laryngeal mask airway in children British Journal of Anaesthesia 2001 86 1 122 124 10.1093/bja/86.1.122 11575387

[20] Mizushima A Wardall GJ Simpson DL The laryngeal mask airway in infants Anaesthesia 1992 47 849 51 10.1111/j.1365-2044.1992.tb03144.x 1443475

[21] Michalek P Donaldson W Theiler L The use of the i-gel in anaesthesia e Facts and fiction in 2013 Trends in Anaesthesia and Critical 2013 3 5 246 251 10.1016/J.TACC.2013.06.002

[22] Theiler L Gutzmann M Kleine-Brueggeney M Urwyler N Kaempfen B i-gel^TM^ supraglottic airway in clinical practice: a prospective observational multicentre study British Journal of Anaesthesia 2012 109 6 990 995 10.1093/bja/aes309 22956643

[23] Ajithan SE Puri A Kapoor MC Comparison of leakage test and ultrasound imaging to validate ProSeal supraglottic airway device placement Journal of Anaesthesiology Clinical Pharmacology 2020 36 2 227 232 10.4103/joacp.JOACP_332_19 33013039 PMC7480306

